# Comparison of the 10-, 14- and 20-Item CES-D Scores as Predictors of Cognitive Decline

**DOI:** 10.3390/brainsci13111530

**Published:** 2023-10-30

**Authors:** Ainara Jauregi-Zinkunegi, Rebecca Langhough, Sterling C. Johnson, Kimberly D. Mueller, Davide Bruno

**Affiliations:** 1 School of Psychology, Liverpool John Moores University, Liverpool L3 3AF, UK; a.jauregizinkunegi@ljmu.ac.uk; 2 Wisconsin Alzheimer’s Institute, School of Medicine and Public Health, University of Wisconsin-Madison, Madison, WI 53705, USA; rekoscik@wisc.edu (R.L.);; 3 Wisconsin Alzheimer’s Disease Research Center, School of Medicine and Public Health, University of Wisconsin-Madison, Madison, WI 53705, USA; 4 Department of Medicine, University of Wisconsin-Madison, Madison, WI 53705, USA; 5 Geriatric Research Education and Clinical Center, William S. Middleton Veterans Hospital, Madison, WI 53225, USA; 6 Department of Communication Sciences and Disorders, University of Wisconsin-Madison, Madison, WI 53705, USA

**Keywords:** depression, CES-D, cognitive decline, MCI, aging

## Abstract

The association between depressive symptomatology and cognitive decline has been examined using the Centre for Epidemiologic Studies-Depression Scale (CES-D); however, concerns have been raised about this self-report measure. Here, we examined how the CES-D total score from the 14- and 10-item versions compared to the 20-item version in predicting progression to cognitive decline from a cognitively unimpaired baseline. Data from 1054 participants were analysed using ordinal logistic regression, alongside moderator and receiver-operating characteristics curve analyses. All baseline total scores significantly predicted progression to cognitive decline. The 14-item version was better than the 20-item version in predicting consensus diagnosis, as shown by their AICs, while also showing the highest accuracy when discriminating between participants by diagnosis at last visit. We did not find sex to moderate the relationship between CES-D score and cognitive decline. Current findings suggest the 10- and 14-item versions of the CES-D are comparable to the 20-item version, and that the 14-item version may be better at predicting longitudinal consensus diagnosis compared to the 20-item version.

## 1. Introduction

Depression is one of the most frequently reported psychiatric disorders in older adults [1] and has been found to affect up to 50% of people living with Alzheimer’s disease (AD) dementia [2,3]. Depressive symptomatology is associated with cognitive decline in elderly individuals (e.g., [4,5,6,7,8,9,10,11,12,13,14]). Moreover, a study of two longitudinal cohorts reported that the presence of persistent or deteriorating depressive symptoms were related to faster cognitive decline, even among individuals with mild symptoms [15]. Depressive symptoms have also been shown to be associated with a greater risk of developing mild cognitive impairment (MCI, e.g., [16,17,18,19]), AD, or dementia in older adults (e.g., [2,9,14,20,21,22,23,24,25,26]). A cohort study using data showed that depression increased the risk of dementia by from 10 to 20 times in the first year after depression diagnosis, and the risk persisted even 20 years or more after the diagnosis [27]. However, contradictory findings have also been reported, indicating that these symptoms do not significantly increase the risk of cognitive decline (e.g., [28,29,30,31]) or dementia (e.g., [32,33,34]). Various factors might contribute to the disparity in results, including participants’ age, duration of follow-up, and methods used to diagnose AD or assess depressive symptoms [26]. Overall, most studies support this association (for a review, see [35,36]), although the nature of the relationship remains unclear [37].

One of the most popular screening tools for depressive symptomatology is the Centre for Epidemiologic Studies-Depression Scale (CES-D; [38]). Although the CES-D is comparable to the Beck Depression Inventories [39,40] and widely used in clinical and research studies [41], it has been described as too long [42] or challenging [43] when used in certain populations [44], such as in people with cognitive impairment or with poorer literacy. The item content of the original 20-item CES-D has also been questioned, as certain items have been found to perform differently depending on sex, age, health, cultural, and/or social differences [41,45]. Items related to social issues, such as “People were unfriendly” and “I felt that people disliked me”, could be measuring other constructs, such as perceived social skills and symptoms of interpersonal disorders [41,42,46,47,48,49]. Items referring to somatic physical symptoms, “I felt that everything I did was an effort”, could misrepresent depressive symptoms in older individuals [41,50] or in those with chronic pain [41,51]. Moreover, responses to item number 17, “I had crying spells”, have been shown to vary with sex and to result in an increase in total score in women [52,53], causing an overestimation of depressive symptoms in women and an underestimation in men [41]. In a study by Carleton et al. [41], confirmatory factor analyses were conducted to compare previous and new models of the CES-D, and results supported a 14-item, 3-factor model, comprising somatic symptoms, negative affect, and anhedonia; the authors argue the 14-item version (CESD-14) is more consistent with current diagnostic criteria for depression. A 10-item version (CESD-10)**,** in which redundant items are removed [54] and the focus is placed on affective symptoms by reducing somatic items [55], has also been used in studies investigating depression and cognitive decline (e.g., [12,56]).

Although CESD-10 and CESD-14 have been used and validated in previous studies (e.g., [57,58,59,60]), to our knowledge, it is yet unclear how the 10-item and 14-item versions compare to the 20-item version in predicting progression from a cognitively unimpaired baseline to a clinically diagnosed impaired status in older adults. Considering that depressive symptoms may not only serve as a risk factor for cognitive decline but may also be an early sign of cognitive decline [36], the detection of a modifiable risk factor such as depression is crucial, as it provides a better understanding of the predictive abilities of widely used depression screening tools.

The aims of this study were to determine if baseline total CES-D scores were associated with progression to cognitive decline from a cognitively unimpaired baseline, and to examine how the CES-D total score from the 14- and 10-item versions compared to the 20-item version in predicting progression. We tested if the risk of progression differed by sex by conducting a moderator analysis, while also comparing the three CES-D versions. Finally, we examined how the CES-D total score from the 14- and 10-item versions compared to the 20-item version in differentiating between participants who remained cognitively unimpaired stable and those who had cognitively declined at the last follow-up visit. We predicted that baseline total CES-D scores would be associated with progression to cognitive decline, that the 14- and 10-item total scores would be better predictors and discriminators than the original 20-item version, and that the risk of progression would differ between males and females.

## 2. Methods

We report how we determined our sample size, all data exclusions, all manipulations, and all measures in the study. This study was not preregistered.

### 2.1. Participants

The Wisconsin Registry for Alzheimer’s Prevention (WRAP) study is an ongoing longitudinal cohort study based at the University of Wisconsin–Madison, USA, of older adults who attend regular visits; the first follow-up occurs at least after 4 years and then every 2 years (for details, see [61,62]). Participants were classified after each study visit as cognitively unimpaired—stable (CUS), cognitively unimpaired—declining (CUD), MCI, or Dementia, via a consensus conference diagnosis (as described in [63] and in Procedure). For the present study, participants were selected based on their having completed at least two visits with item-level CES-D data, being classified as CUS at CES-D baseline, and either being classified as still cognitively unimpaired (stable or declining), with MCI, or with Dementia at their last visit. From the total pool of 1670 participants, 1054 participants fulfilled the above inclusion criteria: 1019 were native English speakers, 8 were Spanish native speakers, 9 spoke other languages (unspecified), and 18 did not report their native language; 7 participants reported their race as American Indian or Native American, 2 as Asian, 26 as Black or African American, 10 as Spanish or Hispanic, 1008 as White, and 1 as unknown. Item-level data availability for the CES-D began at visit 2, which was considered the baseline for these analyses. At their last visit, from the same participants, 952 individuals were classified as CUS, 79 as CUD, 19 as MCI, and four as dementia. All activities for this study were approved by the institutional review board of the University of Wisconsin–Madison and completed in accordance with the Helsinki Declaration. All participants provided informed consent before testing.

### 2.2. Procedure

At each study visit, participants completed self-report questionnaires on demographics, health history and lifestyle, in addition to clinical assessments, and a neuropsychological test battery (for a full list of procedures and tests, see [61]). To classify individuals based on their cognitive status, WRAP uses a two-tiered consensus conference approach (see [63] for details). Briefly, in the first step, an algorithm that identifies cases where impairment may exist is applied, based on whether or not they meet one or more of the following criteria: (1) the participant obtains 1.5 SDs below the mean on factor scores or individual measures of memory, executive function, language, working memory, or attention [64,65]; (2) cognitive performance on one or more tests fell below values used in other studies as cut-points for clinical MCI diagnoses (e.g., WMS-R Logical Memory II, [66]: story A score <9: AD Neuroimaging Initiative, [67]); or (3) an abnormal informant report indicating subjective cognitive or functional decline. Second, consensus diagnoses of cognitively unimpaired, MCI and dementia are then determined by a team that includes physicians, clinical neuropsychologists, and clinical nurse practitioners, based on cognitive, medical history, lifestyle, subjective cognitive complaints, and informant data, for each visit. The MCI diagnosis follows the core clinical criteria (excluding biomarkers) from Albert et al. ([68], but see also [69]), adopted by the National Institute on Aging (NIA)–Alzheimer’s Association, while dementia diagnosis follows the recommendations from McKhann et al. [70]. If the consensus review committee determines MCI and dementia are absent, the CUD label is assigned when the consensus review team interprets the low performance as indicative of concerning subclinical decline from premorbid levels.

### 2.3. Assessment of Depressive Symptoms

The Center for Epidemiologic Studies Depression Scale (CES-D; [38]) measures levels of depression symptoms experienced in the past week with items expressed as self-statements (e.g., “I talked less than usual”); see Table 1 for a list of items. The original CES-D version contains 20 items, in which participants are asked to indicate the frequency of the symptoms using a scale of 0 (rarely or none of the time, less than 1 day), 1 (some or a little of the time, 1–2 days), 2 (occasionally or a moderate amount of time, 3–4 days), or 3 (most or all of the time, 5–7 days), against a timeframe of the past week. The total score is computed by adding the points from each item, except positive items 4, 8, 12, and 16, for which the scoring is reversed. The 14-item version (CESD-14, [41]) excludes items 9, 10, 13, 15, 17 and 19, whereas the 10-item version (CESD-10, [54]) excludes items 2, 3, 4, 9, 13, 15, 16, 17, 18, and 19; in both versions, the total score is calculated in the same way as the 20-item version. In the current study, the recording of item-level CES-D data began at visit 2, which was considered as a baseline for the statistical analyses. Internal consistency for the 10-item CES-D (Cronbach’s α = 0.80), 14-item CES-D (Cronbach’s α = 0.85), and 20-item CES-D (Cronbach’s α = 0.87) in the current sample was acceptable.

### 2.4. Assessment of Control Variables

Included demographic factors were age at last follow-up visit, sex, and education, while also accounting for the time elapsed between baseline and last follow-up assessment. An *APOE* risk score was calculated based on the odds ratios of the presence of apolipoprotein E genotype (e2/e3/e4 genotype), as previously reported [71], which was included as a covariate. Because vascular factors have been suggested to be linked to late-life depression, cognitive decline, and risk for AD [72,73,74,75,76], the following vascular risk factors were included as covariates: waist–hip ratio (calculated from measurements taken at baseline visit), current smoking, history of diabetes, hypertension, heart disease, and high cholesterol (all dichotomised into yes or no, and assessed via questionnaire). For more details on vascular risk factors and prediction of AD, see Reitz et al. [77].

### 2.5. Statistical Analysis

We ran Mann–Whitney tests or *t*-tests where appropriate to determine if there were differences between participants classified by last cognitive status on the sample characteristics, *APOE* risk score, vascular risk factors, and in the baseline 10-item, 14-item, and 20-item total CES-D scores. See Table 2 for sample details reported for the whole sample and by last cognitive status. To understand how correlated the total scores from the three CES-D versions were, we ran bivariate Spearman’s rank-order correlations between the scores from the 10-, 14-, and 20-item versions of the CES-D. Bivariate correlations were also conducted between the total CES-D scores and the control variables to check for multicollinearity.

To test if baseline total CES-D scores are associated with progression to cognitive decline at last follow-up visit (see Table 3 for sample details at last visit), we conducted three separate ordinal logistic regression analyses (one each for the three CES-D versions). We used either baseline total 10-, 14-, or 20-item score as predictor in each model, follow-up consensus diagnosis as outcome (cognitively unimpaired stable, cognitively unimpaired declining, or clinically diagnosed impaired, by combining MCI and dementia), age at last follow-up assessment, sex, elapsed time between baseline and last follow-up assessment, years of education, *APOE* risk score, current smoking status, history of diabetes mellitus, hypertension, heart disease, high cholesterol and waist–hip ratio as covariates. To determine if the 10- and 14-item CES-D scores are better predictors of risk of progression to cognitive decline than the 20-item scores, we compared AIC fit statistics [78] across otherwise parallel models; lower AIC values indicate a better fit, and a model with a delta-AIC (i.e., the difference between the two AIC values being compared) greater than 2 is considered significantly better than the model to which it is being compared [79].

To test our hypothesis that the risk of progression to CUD or worse impairment at last visit that is associated with baseline CES-D differs between males and females, we examined baseline total CES-D score by sex interactions in ordinal logistic regression models (one each for the 10-, 14- and 20-item version). The included predictors were sex, total CES-D scores (10-, 14- and 20-item in separate models), and the sex * total CES-D scores interaction term, while the outcome and covariates remained the same. If the interaction terms were found to be significantly associated with progression to CUD or worse impairment at last visit, the models were then assessed using a likelihood ratio chi-square test to examine if the full models with the interaction term decreased the deviance over the full models with no interaction term.

We generated receiver-operating characteristic (ROC) curves to check for the sensitivity, specificity, positive predictive value (PPV), and negative predictive value (NPV), of the 10-item, 14-item, and 20-item total CES-D scores. The Area Under the ROC curve (AUC) of each total score was also computed to measure how well the total score of each version can distinguish between cognitively declined participants from those who were CUS at both CES-D baseline and last follow-up visit, where a larger area indicates better performance [80]. The AUCs of the 10-item, 14-item and 20-item total CES-D scores were compared using the Z statistic, and because the ROC curves are expected to be correlated, a nonparametric approach proposed by DeLong et al. [81] was used. A two-sided *p*-value < 0.05 was considered statistically significant and the optimal cut-off point was identified based on the maximum Youden’s index [82]. ROC analyses were performed using MedCalc, version 20.114 (MedCalc Software, Ostend, Belgium).

## 3. Results

**Figure 1:**
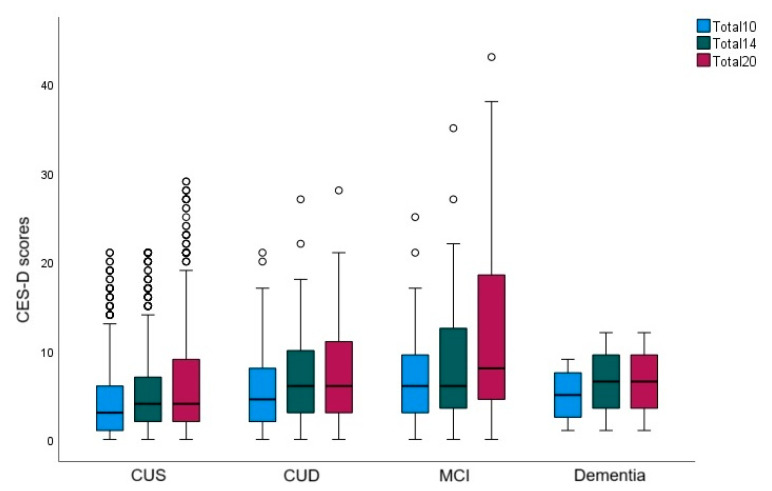
**Boxplot**: Baseline total 10-, 14-, and 20-item CES-D scores by each of the four cognitive statuses at last follow-up visit. CUS: cognitively unimpaired-stable; CUD: cognitively unimpaired-declining; MCI: mild cognitive impairment.

After a mean of 7 years (range = 0−12 years), of the 1054 participants included in the current study, 102 (9.7%) progressed to CUD (79), MCI (19), or dementia (4), whereas 952 (90.3%) remained cognitively unimpaired and stable at last follow-up. Table 2 describes the sample demographic characteristics, *APOE* risk score, vascular risk factors, and baseline total 10-, 14-, and 20-item CES-D scores, for the whole sample and by cognitive status at last follow-up assessment; the table also reports how participants classified by last cognitive status differ on these variables. Figure 1 reports baseline total 10-, 14-, and 20-item CES-D scores by each of the four cognitive statuses at last follow-up visit.

We ran bivariate Spearman’s rank-order correlations between the total scores from the three CES-D versions to understand how they are associated with each other, and between all the variables included in the analyses to check for multicollinearity. The scores from 10-item version were significantly correlated with those from the 14-item version (*r*_s_ = 0.955, *p* < 0.001) and with the scores from the 20-item version (*r*_s_ = 0.943, *p* < 0.001), which were, in turn, mutually correlated (*r*_s_ = 0.979, *p* < 0.001). Although significant associations were found between the rest of the variables, these were either very weak (*r*_s_ = 0.00–0.19) or weak (*r*_s_ = 0.20–0.39); see Table 3 for details.

To test if baseline total CES-D scores were associated with progression to cognitive decline (CUD, MCI and Dementia) from a cognitively unimpaired and stable status, ordinal logistic regression analyses were conducted for each of the three total CES-D scores. The three logistic regression models with either 10-item, 14-item or 20-item total CES-D scores were statistically significant, as were the coefficients of each total CES-D score; see Table 4 for details. Specifically, a one-point increase in baseline total CES-D score from the 10-item version was significantly associated with an increase in the odds of future cognitive decline (b = 0.088; SE = 0.022; *p* < 0.001; OR = 1.092; 95% CI, 1.046–1.141), as was a one-point increase in baseline total CES-D score from the 14-item version (b = 0.067; SE = 0.016; *p* < 0.001; OR = 1.070; 95% CI, 1.036–1.104), and one-point increase in baseline total CES-D score from the 20-item version (b = 0.051; SE = 0.013; *p* < 0.001; OR = 1.052; 95% CI, 1.025–1.080).

To test the hypothesis that the 10- and 14-item CES-D scores are better predictors of risk of progression to cognitive decline than the 20-item scores, we then compared AIC fit statistics across otherwise parallel models. The model with lowest AIC was the baseline total CES-D scores from the 14-item version (AIC = 733.00), closely followed by the model with baseline total scores from the 10-item version (AIC = 733.77) and, finally, the model with baseline total scores from the 20-item version (AIC = 735.07); delta-AIC between the 20-item model and the 14-item model was greater than 2, indicating there was a significant difference between them, but not between the 10- and 20-item models or the 10- and 14-item models

To test our hypothesis that the risk of progression to CUD or worse impairment at last visit that is associated with baseline CES-D differs between males and females, we computed an interaction term with baseline total CES-D score and sex (one each for the 10-, 14- and 20-item version), and compared the interaction models to the non-interaction models using a likelihood ratio test. The three regression models with the interaction term were statistically significant, but their interaction terms were not (total 10-item score by sex, b = −0.004; SE = 0.049; *p* = 0.934; total 14-item score by sex, b = −0.016; SE = 0.036; *p* = 0.660; total 20-item score by sex, b = −0.011; SE = 0.030; *p* = 0.701); thus, changes in model deviance were not tested further.

**Figure 2:**
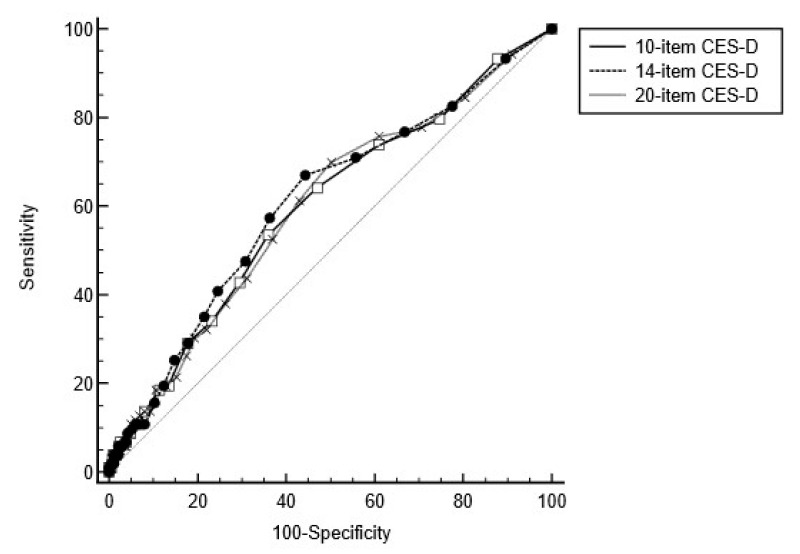
Receiver Operating Characteristic (ROC) curves of the 10-item, 14-item, and 20-item total CES-D scores. Line with the squares refers to the 10-item CES-D, circles to the 14-item CES-D and crosses to the 20-item CES-D.

To investigate how the CES-D total score from the 14- and 10-item versions compared to the 20-item version in differentiating between cognitive declined and cognitively unimpaired and stable participants at last follow-up visit, ROC analyses were conducted. The ROC curve analyses showed that the 10-item (Z = 3.217; *p* = 0.001), 14-item (Z = 3.593; *p* = 0.000), and 20-item (Z = 3.219; *p* = 0.001) total scores of the CES-D significantly discriminated between cognitively declined participants and those who were cognitively unimpaired and stable at last follow-up visit; see Figure 2 for ROC curves. The AUCs showed that the 14-item total score had the highest accuracy (60.8%; SE = 0.03; 95% CI, 57.8–63.8%), followed by the 10-item score (59.6%; SE = 0.03; 95% CI, 56.6–62.6%), and the 20-item score (59.6%; SE = 0.03; 95% CI, 56.6–62.6%). Z tests indicated that the 14-item and 20-item AUCs were significantly different (Z = 2.123; *p* = 0.034), whereas the 10-item and 20-item AUCs were not (Z = 0.014; *p* = 0.989), nor were the 10-item and 14-item AUCs (Z = 1.182; *p* = 0.237). 

As the three versions of total CES-D scores significantly differentiated between the two groups, diagnostic concordance was assessed using positive predictive values (PPV) and negative predictive values (NPV). For the 10-item total score, with a cut-off of 4 based on the Youden index, the PPV was 13.9% and the NPV was 92.7% (sensitivity 53.40%, specificity 64.08%). For the 14-item total score, a cut-off of 4 produced a PPV of 14.1% and a NPV of 94.0% (sensitivity 66.99%, specificity 55.67%). Finally, for the 20-item total score, a cut-off of 5 produced a PPV of 13.1% and a NPV of 93.9% (sensitivity 69.90%, specificity 49.79%).

## 4. Discussion

In the current study, we investigated if baseline total CES-D scores were associated with progression to cognitive decline from a cognitively unimpaired baseline and assessed how the CES-D total score from the 14- and 10-item versions compared to the 20-item version in predicting progression. We also tested if the risk of progression differed by sex by conducting a moderator analysis, while comparing the three CES-D versions. Lastly, we compared the CES-D total score from the 14- and 10-item versions to the 20-item version, in their ability to differentiate between participants who had progressed to cognitive decline from those who had not at last follow-up visit.

In contrast to the few studies reporting that depressive symptoms are not associated with future cognitive decline (e.g., [28,29,30,31]), our ordinal logistic regression analyses showed that separate models with baseline total CES-D scores from either the 10-item, 14-item, or 20-item version significantly predicted follow-up consensus diagnosis (cognitively unimpaired—stable vs. cognitive unimpaired—declining, MCI, or dementia) after approximately 7 years whilst controlling for age at last follow-up assessment, elapsed time between baseline and last follow-up assessment, years of education, APOE risk score, and vascular factors. These findings are in line with most studies that reported an association between depressive symptoms and subsequent cognitive decline in older individuals [4,5,6,7,8,9,10,11,12,13,14], even though the nature of the relationship remains unclear [37] and requires further investigation.

Revised versions of the CES-D, such as the 14-item [41] or the 10-item version [54], have been proposed to address concerns regarding item content (e.g., gender bias), latent factor structure, or the length of the original 20-item scale [41,44]. However, it is not known how the 10-item and 14-item versions compare to the original 20-item scale in predicting progression to cognitive decline in older adults. The current results showed that the variance explained by the models with the 10-item or 14-item total CES-D scores was comparable to the variance explained by the 20-item model in predicting progression to cognitive decline. The AICs from the models with the 10-item and 14-item version scores were similar, indicating a difference between them of 0.77 AIC units, while the model with the 20-item version showed the highest AIC of the three, with the difference being greater than 2 AIC units when compared to the 14-item version, but not between the 20- and 10-item versions; the difference between these two versions was 1.30 AIC units. Following the rule-of-thumb requiring a difference of at least two AIC units to conclude that model fit is better, we suggest the 14-item version may be better at predicting longitudinal consensus diagnosis compared to the 20-item version. These findings leave the 10-item version in apparent limbo, as that version is not significantly better than the 20-item version, nor is it significantly worse than the 14-item version. This apparent contradiction suggests that, while the 10-item version is numerically a better test to predict diagnosis than the 20-item version, we do not have firm enough evidence to draw that conclusion. Considering that depressive symptoms may be an early sign of cognitive decline [36], current results showing the longitudinal predictive abilities of the three CES-D versions, and especially the 14-item version, provide further support for their use.

As rates of dementia and depression, along with depressive symptoms’ profiles and clinical course, are known to differ between the sexes [83,84], it could be argued that sex should be considered when assessing risk factors that can be modified [37], as is the case with depressive symptomatology. To better understand if sex moderates the relationship between depressive symptoms and risk of progression to CUD or worse impairment, we also examined baseline total CES-D score by sex interactions. Our results did not show the interaction term to be a significant predictor of progression to cognitive decline.

We also examined whether the total CES-D scores differentiated between cognitively unimpaired participants who remained stable and those who had cognitively declined at last follow-up visit, and ROC curve analyses indicated that the total scores of the CES-D from the three versions significantly discriminated between them. When comparing the accuracy of each of the three CES-D versions, we found that the total score from the 14-item version showed the highest accuracy, while the 10- and 20-item tests AUCs were identical. This latter finding is in line with the results of Chen and Chan [43], who reported that the 10-item version was comparable to the original version in screening for depressive symptoms in elderly participants with mild dementia. Although these results show that the three CES-D versions perform better than chance in differentiating the two groups (i.e., 50%), the AUC values of the 10-item (59.6%), 14-item (60.8%), and 20-item (59.6%) models observed here are very similar and considered “poor”; generally, AUC values between 0.9 and 1 are considered “outstanding”, between 0.8 and 0.9 are considered “excellent”, and values are considered “acceptable” between 0.7 and 0.8. It is possible that, within the cognitively declined group, the difference in the number of participants who were cognitively unimpaired but declining (N = 80) and those who had MCI (N = 19), or dementia (N = 4) was too large. As a result, the ability of the three CES-D versions to discriminate between cognitively unimpaired and stable, and a group largely comprised of participants who were unimpaired but declining, might have been affected.

This study has several strengths, such as the length of the follow-up, with an average of approximately 7 years between the baseline visit and last follow-up visit, and that participants were classified via consensus conference diagnosis, based on cognitive, medical history, lifestyle, subjective cognitive complaints, and informant data, for each visit (see Procedure section for details and [63]). However, this study also has some limitations. The sample size of the cognitively declined group at last follow-up visit was far from ideal; this, however, is not surprising, considering the progression rates of cognitively unimpaired individuals observed in previous studies (progression to CU-D, formerly referred to as early MCI, was 14% in [85]; and 15.2% in [61]). The sample also consisted mostly of White participants, restricting the generalizability of current findings. Finally, it should be noted that current analyses and results were extrapolated from the 20-item version of the CES-D rather than from testing the three versions separately.

In summary, this study investigated if baseline total CES-D scores were associated with progression to cognitive decline from a cognitively unimpaired baseline, assessed how the CES-D total score from the 14- and 10-item versions compared to the 20-item version in predicting progression, CES-D by sex interaction, and the accuracy of baseline CES-D scores to discriminate between participants who had cognitively declined from those who remained unimpaired and stable at last visit. The results showed that baseline total scores from the three CES-D versions significantly predicted progression, and that the model with total scores from the 14-item version was better than the model with scores from 20-item version in predicting progression to cognitive decline, as shown by their AICs. We did not find sex to moderate the relationship between CES-D score and cognitive decline. The 14-item baseline total CES-D score also showed the highest accuracy when discriminating between those who were cognitively unimpaired and stable and those who had cognitively declined at last follow-up visit. We believe that the 14-item version of the CES-D could be a good alternative to the original 20-item version for studies investigating depressive symptoms and cognitive decline.

## Figures and Tables

**Table 1:** The 20 items from the original CES-D, items included in the CES-D 10 and CES-D 14 versions. X denotes item is included.

20-Item CES-D	10-Item CES-D	14-Item CES-D
1. I was bothered by things that usually don’t bother me.	X	X
2. I did not feel like eating; my appetite was poor.		X
3. I felt that I could not shake off the blues, even with help from my family or friends.		X
4. I felt I was just as good as other people.		X
5. I had trouble keeping my mind on what I was doing.	X	X
6. I felt depressed.	X	X
7. I felt that everything I did was an effort.	X	X
8. I felt hopeful about the future.	X	X
9. I thought my life had been a failure.		
10. I felt fearful.	X	
11. My sleep was restless.	X	X
12. I was happy.	X	X
13. I talked less than usual.		
14. I felt lonely.	X	X
15. People were unfriendly.		
16. I enjoyed life.		X
17. I had crying spells.		
18. I felt sad.		X
19. I felt that people disliked me.		
20. I could not get “going”.	X	X

**Table 2:** Sample characteristics and comparison among participants by their last cognitive status. Means (standard deviations) or number of participants (percentages) are reported for the variables included in the regression analyses by whole sample and consensus diagnosis at last follow-up assessment. For heart disease, diabetes mellitus, hypertension, and high cholesterol, yes refers to a previous or current history of the disease.

Characteristic	Total	Cognitively Unimpaired Stable	Cognitively Unimpaired Declining	MCI or Dementia	*p*
No. participants	1054	952 (90.3%)	79 (7.5%)	23 (2.2%)	
Age at last visit	64.65 (6.9)	64.32 (7.0)	67.46 (5.7)	68.65 (5.9)	**0.001**
Gender (females)	766 (72.7%)	697 (73.2%)	52 (65.8%)	17 (73.9%)	0.364
Education (years)	16.27 (2.8)	16.32 (2.8)	16.20 (2.6)	14.39 (2.2)	**0.004**
APOE risk score	1.18 (0.7)	1.14 (0.7)	1.40 (0.8)	1.67 (0.9)	**0.001**
Elapsed time	6.86 (3.1)	6.85 (3.1)	7.16 (2.5)	6.17 (2.6)	0.375
Heart disease (yes)	47 (4.5%)	40 (4.2%)	3 (3.8%)	4 (17.4%)	**0.010**
Diabetes mellitus (yes)	63 (6.0%)	52 (5.5%)	8 (10.1%)	3 (13.0%)	0.086
Hypertension (yes)	261 (24.8%)	221 (23.2%)	29 (36.7%)	11 (47.8%)	**0.001**
High cholesterol (yes)	457 (43.4%)	403 (42.3%)	36 (45.6%)	18 (78.3%)	**0.002**
Baseline smoking (yes)	449 (42.6%)	405 (42.5%)	36 (45.6%)	8 (34.8%)	0.651
Baseline WHR	0.86 (0.1)	0.86 (0.1)	0.88 (0.8)	0.88 (0.9)	0.255
Baseline CESD-10	4.53 (4.2)	4.38 (4.1)	5.46 (4.4)	7.35 (6.6)	**0.001**
Baseline CESD-14	5.76 (5.7)	5.55 (5.5)	7.24 (6.1)	9.13 (9.0)	**0.001**
Baseline CESD-20	6.82 (6.9)	6.59 (6.7)	8.24 (7.3)	11.26 (11.5)	**0.001**

Notes: *p*-values from *t*-tests or Mann–Whitney tests where appropriate. WHR = waist–hip ratio. Bold format—statistically significant.

**Table 3:** Bivariate Spearman’s rank-order correlations. Correlation coefficients between the variables included in the regression analyses.

Variable	1	2	3	4	5	6	7	8	9	10	11	12	13
1. Age at last visit	1	−0.049	0.041	−0.092 **	0.101 **	0.023	0.187 **	0.046	0.138 **	0.074 *	−0.076 *	−0.089 **	−0.107 **
2. Gender		1	−0.117 **	0.006	−0.094 **	−0.016	0.071 *	0.016	−0.052	0.102 **	0.115 **	0.093 **	0.101 **
3. Education years			1	−0.014	−0.043	−0.009	−0.078 *	−0.079 *	−0.066 *	−0.140 **	−0.063 *	−0.069 *	−0.073 *
4. APOE risk score				1	−0.032	0.001	−0.046	−0.028	0.109 **	−0.038	0.011	0.007	0.013
5. Heart disease					1	0.023	0.100 **	0.102 **	0.061 *	0.024	0.004	−0.015	−0.023
6. Diabetes mellitus						1	0.161 **	−0.031	0.151 **	0.141	0.006	0.015	0.013
7. Hypertension							1	0.013	0.217 **	0.160 **	0.062 *	0.048	0.051
8. Current smoking								1	0.017	0.076 *	0.075 *	0.074 *	0.070 *
9. High cholesterol									1	0.161 **	0.060	0.068 *	0.067 *
10. Waist–hip ratio										1	0.057	0.067 *	0.068 *
11. CESD-10											1	0.955 **	0.943 **
12. CESD-14												1	0.979 **
13. CESD-20													1

Note: For heart disease, diabetes mellitus, hypertension, and high cholesterol, answers were dichotomized into yes/no, for previous or current history of the disease. CESD-10, CESD-14, and CESD-20, refer to baseline total scores from the 10-, 14-, and 20-item versions of the CES-D. * *p* < 0.05; ** *p* < 0.01.

**Table 4:** Ordinal logistic regression models predicting progression from CUS to CUD or clinically diagnosed impaired status (MCI and Dementia combined) at last follow-up visit.

Measures	10-Item Model ^1^		14-Item Model ^2^		20-Item Model ^3^	
	OR (95%CI)	*p*	OR (95%CI)	*p*	OR (95%CI)	*p*
Age last visit	1.10 (1.06–1.14)	**0.000**	1.10 (1.06–1.14)	**0.000**	1.10 (1.06–1.14)	**0.000**
Gender	0.63 (0.39–1.00)	0.052	0.63 (0.39–1.01)	0.054	0.63 (0.39–1.01)	0.055
Elapsed time	0.97 (0.90–1.05)	0.448	0.97 (0.90–1.05)	0.457	0.97 (0.90–1.05)	0.450
Education	0.93 (0.85–1.01)	0.072	0.93 (0.85–1.01)	0.076	0.93 (0.85–1.01)	0.078
APOE score	1.92 (1.47–2.51)	**0.000**	1.91 (1.47–2.50)	**0.000**	1.91 (1.46–2.49)	**0.000**
Heart	1.19 (0.50–2.82)	0.692	1.18 (0.49–2.82)	0.708	1.20 (0.51–2.86)	0.675
Diabetes	1.57 (0.74–3.34)	0.238	1.59 (0.75–3.38)	0.225	1.57 (0.74–3.33)	0.238
Hypertension	1.65 (1.03–2.64)	**0.037**	1.66 (1.04–2.66)	**0.034**	1.65 (1.03–2.64)	**0.036**
Smoking	0.88 (0.57–1.36)	0.579	0.87 (0.57–1.35)	0.544	0.88 (0.57–1.35)	0.551
High cholest.	0.97 (0.62–1.52)	0.897	0.97 (0.62–1.53)	0.907	0.97 (0.62–1.52)	0.894
WHR	1.06 (0.82–1.38)	0.639	1.06 (0.82–1.37)	0.650	1.07 (0.82–1.38)	0.631
CESD score	1.09 (1.05–1.14)	**0.000**	1.07 (1.04–1.10)	**0.000**	1.05 (1.03–1.08)	**0.000**
AIC	733.77		733.00		735.07	

Note: β (95%CI) = Odds ratio (95% confidence interval). AIC = Akaike Information Criterion. Heart = heart disease. High cholest. = high cholesterol. WHR = waist–hip ratio. ^1^ Model with 10-item Total CES-D score: χ^2^(12) = 76.026, *p* < 0.001, Nagelkerke R^2^ = 13.3%. ^2^ Model with 14-item Total CES-D score: χ^2^(12) = 77.305, *p* < 0.001, Nagelkerke R^2^ = 13.5%. ^3^ Model with 20-item Total CES-D score: χ^2^(12) = 74.900, *p* < 0.001. Nagelkerke R^2^ = 13.1%. Bold format—statistically significant.

## Data Availability

Data can be requested here: https://wrap.wisc.edu/data-requests.
